# Protocol for diaphragm pacing in patients with respiratory muscle weakness due to motor neurone disease (DiPALS): a randomised controlled trial

**DOI:** 10.1186/1471-2377-12-74

**Published:** 2012-08-16

**Authors:** Christopher J McDermott, Chin Maguire, Cindy L Cooper, Roger Ackroyd, Wendy O Baird, Simon Baudouin, Andrew Bentley, Stephen Bianchi, Stephen Bourke, Mike J Bradburn, Simon Dixon, John Ealing, Simon Galloway, Dayalan Karat, Nick Maynard, Karen Morrison, Naveed Mustfa, John Stradling, Kevin Talbot, Tim Williams, Pamela J Shaw

**Affiliations:** 1 Sheffield Institute for Translational Neuroscience (SITraN), University of Sheffield, 385A Glossop Road, Sheffield, S10 2HQ, UK; 2 Sheffield Clinical Trials Research Unit, University of Sheffield, ScHARR, Sheffield, UK; 3 Sheffield Teaching Hospitals, Northern General Hospital, Sheffield, UK; 4 Research Design Service, Public Health, University of Sheffield, ScHARR, Sheffield, UK; 5 Royal Victoria Hospital, Newcastle Upon Tyne, UK; 6 University Hospital of South Manchester, Manchester, UK; 7 Sheffield Teaching Hospitals, Royal Hallamshire Hospital, Sheffield, UK; 8 North Tyneside General Hospital, North Shields, UK; 9 Health Economics and Decision Science, University of Sheffield, ScHARR, Sheffield, UK; 10 Salford Royal Hospital Foundation Trust, Salford, UK; 11 Oxford Radcliffe Hospitals, Churchill Hospital, Oxford, UK; 12 Queen Elizabeth Hospital, University Hospitals Birmingham, Birmingham, UK; 13 University Hospital North Staffordshire, City General Site, Stoke on Trent, UK; 14 Oxford Radcliffe Hospitals, John Radcliffe Hospital, Headington, Oxford, UK

## Abstract

**Background:**

Motor neurone disease (MND) is a devastating illness which leads to muscle weakness and death, usually within 2-3 years of symptom onset. Respiratory insufficiency is a common cause of morbidity, particularly in later stages of MND and respiratory complications are the leading cause of mortality in MND patients. Non Invasive Ventilation (NIV) is the current standard therapy to manage respiratory insufficiency. Some MND patients however do not tolerate NIV due to a number of issues including mask interface problems and claustrophobia. In those that do tolerate NIV, eventually respiratory muscle weakness will progress to a point at which intermittent/overnight NIV is ineffective. The NeuRx RA/4 Diaphragm Pacing System was originally developed for patients with respiratory insufficiency and diaphragm paralysis secondary to stable high spinal cord injuries. The DiPALS study will assess the effect of diaphragm pacing (DP) when used to treat patients with MND and respiratory insufficiency.

**Method/Design:**

108 patients will be recruited to the study at 5 sites in the UK. Patients will be randomised to either receive NIV (current standard care) or receive DP in addition to NIV. Study participants will be required to complete outcome measures at 5 follow up time points (2, 3, 6, 9 and 12 months) plus an additional surgery and 1 week post operative visit for those in the DP group. 12 patients (and their carers) from the DP group will also be asked to complete 2 qualitative interviews.

**Discussion:**

The primary objective of this trial will be to evaluate the effect of Diaphragm Pacing (DP) on survival over the study duration in patients with MND with respiratory muscle weakness. The project is funded by the National Institute for Health Research, Health Technology Assessment (HTA) Programme (project number 09/55/33) and the Motor Neurone Disease Association and the Henry Smith Charity. Trial Registration: Current controlled trials ISRCTN53817913. The views and opinions expressed therein are those of the authors and do not necessarily reflect those of the HTA programme, NIHR, NHS or the Department of Health.

## Background

Motor Neurone Disease (MND) is the third commonest neurodegenerative disease with an annual incidence of 2-3 in 100,000 and prevalence of 5-8 per 100,000 [1-3]. Patients experience increasing muscle weakness affecting the limbs, speech and swallowing, and breathing. As the diaphragm and intercostal muscles become weak patients experience sleep fragmentation and symptoms of carbon dioxide retention. These consist of early morning headaches, unrefreshing sleep and sleepiness during the day [4,5]. These symptoms severely impact on cognition and quality of life [6]. When respiratory muscle weakness is advanced, patients can be breathless at rest and are prone to recurrent chest infections. Severe respiratory muscle weakness is a poor prognostic sign and once the forced vital capacity (a measure of respiratory muscle strength) reaches less than 50% of the predicted value, mortality at 9 months ranges from 60%-100% [7,8].

An important advance in the management of respiratory symptoms in MND has been the demonstration of the beneficial effects of non invasive ventilation (NIV). A randomised controlled trial demonstrated a median survival benefit of approximately 7 months (p = 0.006), in MND patients using NIV who had good bulbar function [9]. This survival benefit was accompanied by a significant and sustained improvement in quality of life. As experience with NIV has developed, areas of continuing need have been identified which are not sufficiently addressed by NIV alone:

a) MND patients with significant compromise of bulbar function do not tolerate NIV and in the above trial of NIV, no significant survival benefit was demonstrated for this group [9].

b) Similarly some patients fail to tolerate NIV due to claustrophobia and mask interface problems. In addition although the NIV systems are ideal for overnight use, during the day the mask interface can interfere with communication and feeding and the ventilator itself, although small, does restrict mobility.

c) Eventually respiratory muscle weakness progresses to a point at which intermittent/overnight NIV is ineffective.

There is therefore, a need for additional complementary respiratory support to further aid respiratory muscle weakness and so potentially provide a prolongation of good quality life.

Diaphragm pacing (DP) is a technique initially developed for the treatment of respiratory muscle weakness in patients with spinal cord injury [10]. In this patient group it has allowed patients to reduce their time on mechanical ventilation or even remove the need for mechanical ventilation [11]. The NeuRX RA/4 System is a four channel percutaneous neuromuscular stimulation system. Intramuscular electrodes are implanted laparoscopically into the abdominal surface of the diaphragm, with leads tunnelled to an exit site on the abdomen. A small external stimulator delivers the stimulus pulses and provides respiratory timing. It is hypothesised that the potential benefits of DP in MND may come from a restoration of the coordination of respiration, lost as a result of upper motor neurone dysfunction, as well as conditioning of diaphragm muscle. In a healthy diaphragm slow twitch type I muscle fibres predominate. Disuse and suppression of the diaphragm activity, due to artificial ventilation, has been demonstrated to lead quickly to atrophy and to a predominance of fast type IIb muscle fibres [12]. The predominant type IIb muscles fibres lead to inefficient uncoordinated diaphragm contractions. In MND a similar process is likely to occur due to disuse (secondary to UMN dysfunction) and suppression of diaphragm activity due to NIV. DP may condition the diaphragm with a conversion back to efficient type I muscle fibres [13].

The anticipated benefits of DP in the MND patient group are: improved survival; improved quality of life; reduction in need for NIV; a less intrusive method of providing respiratory support compared to NIV.

### Diaphragm pacing

The NeuRx R/A4 device, has been utilized in over 350 patients to date, including two separate investigational device exemption (IDE) trials [14]. There are over 275 patient years of cumulative use of the device with the initial spinal cord injured (SCI) patient utilizing the device continuously for 10 years.

### Efficacy evidence

A pilot feasibility study of 16 patients with MND implanted with DP demonstrated provisional safety and tolerability data and a decline in forced vital capacity of 0.9% per month following implantation, compared to 2.4% per month before the procedure [13]. One hundred and six MND patients have been implanted with the NeuRX RA/4 Device in a U.S. Food and Drug Administration (FDA) prospective multi-centre trial. The planned primary analysis of this study was the change in rate of decline of FVC between lead-in (3 months) and DP treatment (12 months) phases for patients not using NIV. The full study results have not yet been published. However subgroup data are available following approval of DP for MND patients under the FDA Humanitarian Device Exemption (HDE) Programme [14,15]. For this approval, analysis was performed on a subgroup of patients who met the Humanitarian Use Designation population criteria (HUD #10-0242) i.e., MND patients with a stimulatable diaphragm by voluntary contraction or phrenic nerve conduction studies, and who were symptomatic due to chronic hypoventilation (CH). The physiological criteria for CH were FVC < 50%, or MIP <60cm H_2_0, or PaC0_2_ ≥45mmHg, or nocturnal oximetry demonstrating O_2_ saturations of ≤ 88% for 5 consecutive minutes.

The median overall survival in the HUD subgroup (n = 84) was 56 months from disease onset, 39 months from diagnosis and 19 months from implantation of device. As part of the FDA HUD approval, survival of implanted patients was compared with the standard care NIV alone arm in a published study by Lechtzin et al [16]. DPS HUD group patients with an FVC ≥ 45% but ≤ 65% (n = 43) were selected for the comparison. The DPS HUD group has an improved median survival of 16 months from diagnosis and 9 months from initiation of NIV compared to the Lechtzin NIV group.

### Safety and tolerability of DP

A total of 350 implantations in Spinal Cord Injured patients and MND patients have taken place [14]. Generally implantation surgery is well tolerated. Detailed safety data has been published on 51 patients with MND who have undergone the implantation procedure (49 in the FDA trial or pilot and 2 compassionate use cases). In the trial/series the FVC at implantation ranged from 45%-89%, whereas the compassionate cases had an FVC of 26% and 28%. All patients were extubated without difficulty and there was no 0 day or 30 day mortality [17].

Within the HDE approval submission safety analysis was performed on the HUD subgroup (n = 86). There were no reports of serious unanticipated adverse device effects and no reports of any serious adverse effects related to the patients’ use of the device following discharge. There were 3 reports of serious adverse events (3.5%) related to implantation. These were pneumothorax (n = 2) requiring intervention and respiratory failure following surgical complications (n = 1) [14]. These were previously recognised risks of laparoscopic procedures.

Discomfort from pacing was reported in 22 patients (26%). This was described as mild in 20 patients and severe in 2 patients. Resolution was achieved in most cases by adjusting the stimulation parameters.

Mild to moderate infection at the percutaneous exit site was reported (n = 9, 9.3%). These were treated with antibiotics. In two cases the subcutaneous wires needed to be repositioned under local anaesthetic.

No serious adverse events occurred as a result of malfunctioning device components. The following malfunctions were reported; anode breaks (n = 18,21%), anode falling out of body (n = 6,7%), external electrode breaks (n = 28, 33%), broken stimulators (n = 6, 7%) and broken stimulator cables (n = 4, 5%) [14]. In practical terms in MND, malfunctioning components resulted in a loss or diminution of conditioning therapy until the malfunction was corrected. Design improvements have subsequently been implemented in an effort to improve reliability and to simplify malfunction resolution.

The data presented with the HDE approval submission are encouraging with regard to efficacy and safety of DP in MND patients. However, an RCT is needed comparing current standard care alone with DP in addition to standard care. The aim of DiPALS will be to determine whether DP in addition to NIV provides added benefit for patients in terms of survival and quality of life outcomes. We will conduct this trial in compliance with the protocol, GCP and regulatory requirements.

## Methods/design

### Study design

DiPALS is a multi-centre prospective randomised controlled interventional trial. The study has been approved by the Cambridge Central Research Ethics Committee, reference 11/EE/0226.

108 patients will be randomly allocated to receive either standard care (NIV) or standard care with additional DP in 5 centres. The participants will be male or female above the age of 18 yrs. Use of the device in the management of a patient’s respiratory dysfunction (device parameters, frequency and length of sessions) will be managed at all centres. Participants will be requested to attend visits in order to obtain safety, quality of life, survival and health economic follow-up data at 2, 3, 6, 9 and 12 months post randomisation. This is a non commercial, portfolio study supported by the DeNDRoN (Dementia and Neurodegnerative Diseases Clinical Research Network). It is funded by the HTA (Health Technology Assessment) Programme and the Motor Neurone Disease Association and the Henry Smith Charity.

### Primary research objective

The primary objective of this trial will be to evaluate the effect of Diaphragm Pacing (DP) on survival over the study duration in patients with MND/ amyotrophic lateral sclerosis (ALS) with respiratory muscle weakness.

### Secondary research objectives

The secondary objectives of this trial will be to evaluate the effect of Diaphragm Pacing (DP) on:

Quality adjusted life years (QALYs), as calculated by combining EQ-5D and mortality data [18]. The objective of the health economic analysis will be to assess the cost-effectiveness of DP compared to standard care in patients with ALS/MND. A cost-utility analysis will be undertaken using the costs, EQ-5D and mortality data from the trial. This will be supplemented with decision analytic modelling to estimate lifetime cost-effectiveness for the patient cohort recruited to the trial.

Quality of life of the patient, as assessed by the sleep apnoea quality of life index (SAQLI), SF-36 [6,9] and qualitative interviews.

Quality of life of the main carer of the patient, as assessed by the Caregiver Burden Inventory [19] and qualitative interviews.

For each efficacy endpoint, the treatment effect will be assessed by analysing the difference between groups over the 12-month follow-up period, and the difference at 12 months.

**Figure 1:**
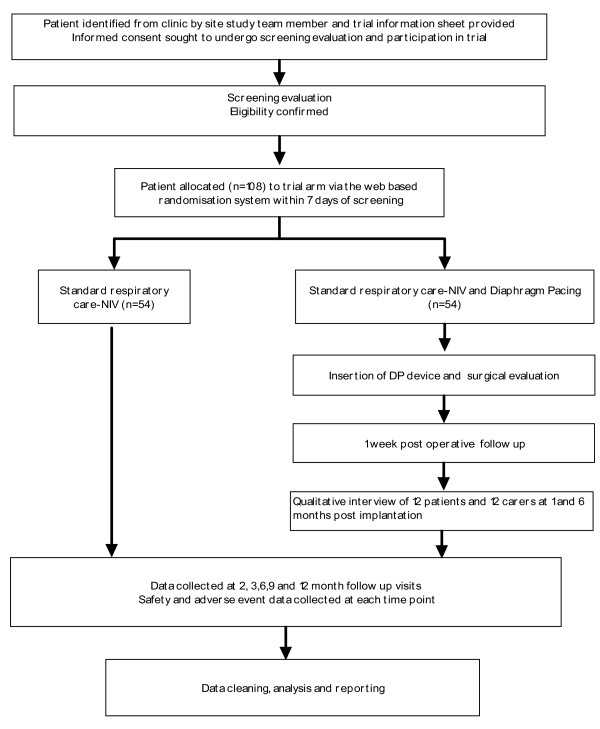
Trial Overview.

The trial will also assess the safety and tolerability of DP. Study endpoints safety (adverse events) and tolerability (patient withdrawal from treatment) will be assessed at each time point over the course of the trial. Please refer to Figure 1: Trial overview.

### Subject population

Participants will be identified by the neurology or respiratory clinicians at each site. Each potential participant will be given a study patient information leaflet which will detail what will happen if they choose to take part.

### Inclusion criteria

Participants aged 18 or older may be recruited onto the trial who have familial or sporadic MND/ALS diagnosed as laboratory-supported probable, probable, or definite according to the World Federation of Neurology El Escorial criteria. Female participants who are currently pregnant or breastfeeding will be excluded. Participants must be stabilised on Riluzole therapy for at least 30 days. Participants must have respiratory insufficiency as determined by one or more of the following criteria:

a) Forced Vital Capacity (FVC) less than 75% predicted

b) Supine Vital Capacity (VC) less than 75% of sitting or standing VC

c) Sniff Nasal Inspiratory Pressure (SNIP) less than 65 cmH2O men, or 55cmH_2_O women in the presence of symptoms

d) Sniff Nasal Inspiratory Pressure (SNIP) less than 40 cmH_2_O

e) PaCO2 > 6kPa (daytime)

f) Significant overnight O2 desaturation (>5% of night with Sp02 <90% during overnight oximetery)

Additionally, an evaluation of bilateral phrenic nerve function will be undertaken to ensure that the participant has a stimulatable diaphragm. Clinical assessment indicating acceptable bilateral phrenic nerve function consists of either absence of paradoxical abdominal wall movement during a sniff maneuver and recording less than 10% decline of FVC when moving from sitting to supine position, OR on ultrasound evidence of at least 1 cm of downward diaphragm movement independent of thoracic or abdominal wall movement during the patient performing a sniff manoeuvre (sharp inhalation through the nose).

### Exclusion criteria

Participants must not have a prior NIV prescription or a pre-existing implanted electrical device such as pacemaker or cardiac defibrillator. Any underlying cardiac, pulmonary diseases or other disorders that would affect pulmonary function tests independently of MND/ALS, would increase the risk of general anaesthesia or adversely affect survival over the course of the study will be excluded. Significant decision making incapacity preventing informed consent by the subject due to a major mental disorder such as major depression, schizophrenia or dementia are exclusions. Others include marked obesity affecting surgical access to diaphragm or significant scoliosis/ chest wall deformity and the involvement in any respiratory trial that can influence the safety or outcome measures of this study within three months of the planned implantation of the device or during the year of follow up. A pre-existing diaphragm abnormality such as a hiatus hernia or paraoesophageal hernia of abdominal contents ascending into the thoracic cavity is an exclusion criterion. Patients with a forced Vital Capacity (FVC) < 50% predicted or SNIP < 30 cmH2O who are unable to perform FVC (bulbar patients) would be excluded – because of potential anaesthetic risk. A patient is eligible for the study if all the inclusion and none of the exclusion criteria are met.

### Recruitment, screening and consent

Potentially eligible MND patients with respiratory insufficiency will be identified by either the neurology or respiratory investigator at the site. This will be either at a clinic or from their clinic database. Patients who are attending a routine clinic appointment will be approached about the study at this appointment with the patient information sheet. Patients identified from the clinic list, who are due to come in for a visit, will be sent an information sheet in the post prior to their appointment. At the appointment the patient will be given an opportunity to discuss the study in more detail and ask any questions. All patients will be given as long as they require to consider the Patient Information Sheet. After this period, patients will be approached either by telephone or in clinic, and the patient will be given the option to give informed consent to the screening procedures and the trial.

Consent will be obtained as either full written consent, verbal consent given or consent given via the use of a communication aid. Where non-written consent is obtained an independent witness will be asked to sign the consent form to verify the consent taken.

If the patient consents to the study a member of the site study team (research nurse, respiratory or neurology consultant) will initiate the screening process. The process will involve assessing patient eligibility both against non-clinical and clinical criteria and obtaining baseline assessments. Patients who decline participation will be asked for a reason for their non-participation to help determine common reasons. This will help aid the recruitment strategy as the trial progresses. We will collect basic details (age, gender, reason for exclusion) on all eligible patients to allow completion of the CONSORT flow chart.

A member of the site study team will use the results of the tests in order to assess patient eligibility against the inclusion and exclusion criteria. Patients meeting all the inclusion and none of the exclusion criteria will proceed to randomisation. A member of the site study team will randomise the participant within a week of screening. The patient will be entered onto the study enrolment log.

### Web based randomisation system

The randomisation system is hosted on a secure virtual server at The University of Sheffield and managed by epiGenesys (a wholly-owned subsidiary of the University). Restricted access via the use of username and passwords is applied to each account. Accounts are assigned specific roles to allow only access to the relevant section of the system to the owner of the account as appropriate. The system records a full audit trail of all activities to discourage improper use and to identify the source of any such use.

### Method of randomisation

**Figure 2:**
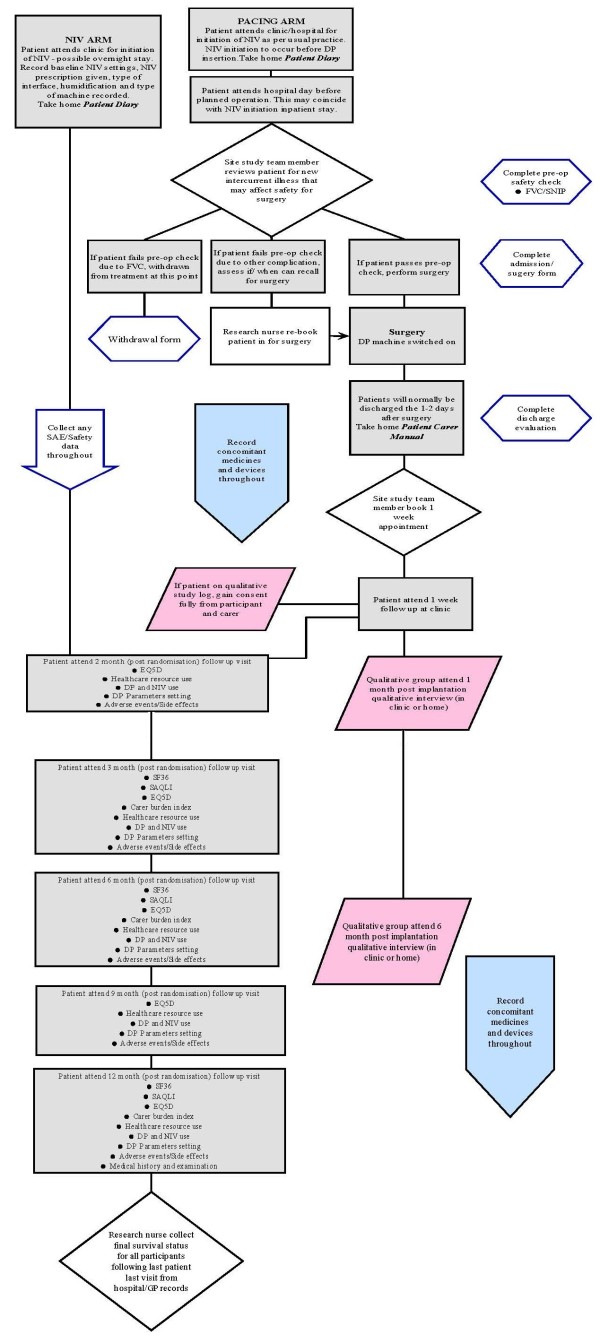
Participant flow within the trial.

Participants will be randomised to either the treatment arm (n = 54) or the control arm (n = 54). Patients will be allocated their treatment by method of minimisation. The minimisation factors will be baseline bulbar function, baseline FVC, age and sex. Patient details (ID, date of birth and the factors above) will be entered into the Sheffield Clinical Trials Research Unit (CTRU) web-based randomisation system and the treatment allocation will be returned. Non-deterministic minimisation will be employed by including a random element into the allocation algorithm. The participant will be informed of their treatment allocation within a week of randomisation either by phone or letter. Please refer to Figure 2 below for the screening and randomisation process.

At randomisation arrangements will be made for both NIV and DP insertion for trial participants. NIV initiation will occur as per usual practice at the study site as soon as possible following randomisation (within 1-2 weeks). For those randomised to the DP arm, a provisional date for implantation will be allocated at randomisation (within 2-3 weeks). Patients should be able to use NIV prior to implantation. NIV should be available to patients in the anaesthetic recovery room post implantation of the device.

### Surgical implantation

In the DP arm participants will be admitted to hospital for insertion of the DP device. A pre-operative safety check will occur either during the admission or in the week leading up to surgery. During the implantation procedure, incisions of 0.5 to 1 inch long will be made in the abdomen. More than one incision will be made so instruments can be passed through the abdominal wall as per standard laparoscopic procedure.

The surgeon will identify the best location to place the electrodes within the diaphragm. A probe will be used to temporarily place an electrode on the surface of the diaphragm and to stimulate the diaphragm muscle at several locations. Once the best locations are identified, the probe will be removed and two electrodes will be placed in each side of the diaphragm muscle. The lead wires from these electrodes will travel under the skin to the abdominal wall. The wires will be trimmed so that the ends sticking out of the skin are only 2 - 6 inches in length. An x-ray will be taken following the surgery to check the position of the wires and to make sure no air has travelled above the diaphragm and into the chest. At the end of surgery the clinical station read out should be printed out displaying a functioning stimulus connection for each electrode wire. This will be used for surgical quality control.

If the damage to the nerve supply to the diaphragm is too great it is possible that the diaphragm will not be able to be stimulated with the electrodes and diaphragm pacing system. The assessments performed during screening are an attempt to determine whether the diaphragm is stimulatable. However, it is only possible to know for sure during the operation. If during the operation it is clear that the diaphragm is not stimulatable then the operation will be stopped and the device will not be inserted.

The training process is simple, as the technique is a modification of a standard abdominal laparoscopic procedure. The clinicians who will be performing this procedure in the treatment arm are experienced surgeons who will all be trained in the DP implantation technique until they are competent to perform the procedure. This training protocol has successfully worked in each of the 15 centres in the FDA study. It is currently standard practice for a member of Synapse Biomedical to be present at surgery when patients are fitted with DP. This will be to provide technical support for the surgical procedure for trial participants.

### Evaluation of electrodes and training

Evaluation of the electrodes and system will be performed prior to discharge from hospital. A system check of the wires will be completed. Electrode evaluation will be performed by adjusting individual stimulus parameters (pulse amplitude, width, and frequency) using the clinical station so that a comfortable level of stimulation can be identified for the diaphragm conditioning sessions. During the initial stimulation period, the participant’s vital signs will be monitored for any abnormalities. The patient will be given a daily target for the number and length of diaphragm pacing sessions. This will be recorded by the study team member in the patient diary.

Training of the participant and their caregiver will take place prior to discharge. This will include instruction in the care and use of the stimulator and data collection in the patient diary. Verbal and written instruction will be provided in a patient/caregiver instruction manual.

Prior to discharge, the participant /or carer must demonstrate proficiency in: cleaning and care of skin, wires and exit site; care and use of the stimulator; attachment & detachment of all components and completion of the Patient Diary.

The initial target for pacing sessions for MND patients is 5 times per day with each session lasting at least 30 minutes. Patients should build up to this target over the first month. In the second month patients should gradually lengthen the training sessions. When using 6-7 hours a day patients should then switch from pacing during the day to using the pacing device overnight whilst asleep. At this stage patients can additionally use the pacing device during the day if they feel benefit but this is not essential. Patients should continue to use their NIV as advised by their study doctor. A Patient Diary will be given to the participant (upon NIV initiation) to take home to record the amount of time spent on DP and/or NIV

### Participant adherence

A member of the site study team will be responsible for data collection at the various time points within the trial. Predominantly the research nurses will be involved in coordinating data collection activities and ensure compliance with appointments. Following surgery a 1 week follow up appointment will be booked for participants in the treatment arm before they leave the hospital. At subsequent follow up time points (2, 3, 6, 9 and 12 months) where possible an appointment for the next time point will be scheduled in. The research nurse will telephone the participant 1 week before the appointment as a reminder where resource allows.

### Withdrawal

Participants are free to withdraw from treatment or the trial at any time. If a participant wishes to withdraw they will be able to speak to a member of the site study team i.e. respiratory or neurology consultants or the research nurse. This will be documented on a participant withdrawal form. Any data already collected during the course of the trial up to the point of withdrawal will be used in the final analysis. We will ask the participant for their permission to continue to collect safety (i.e. adverse event) data and data on survival.

Participants will have three options for study withdrawal: withdraw from treatment but remain within the study (all trial data would continue to be collected at subsequent follow up time points as per protocol); withdraw from study and unless the patient objects, any data collected up to this point would be retained and used in the study analysis and a final survival check will be performed; withdraw from study entirely. Unless the patient objects, any data collected up to this point would be retained and used in the study analysis. If the patient does not wish to be contacted with regard to safety or survival, no further contact relating to the study will be made.

### Qualitative interviews

The qualitative component will draw directly upon both patient and carer’s own experience and views, within the context of everyday lives [20,21]. An essential part of DiPALS is not only to demonstrate the efficacy of DP but also to ensure that any extension of life is not to the detriment of quality of life. The qualitative component will complement the data collected by SF36 and SAQLI for this purpose. The qualitative component will provide information not easily obtained from questionnaires that will facilitate the implementation of respiratory care pathways incorporating Diaphragm pacing should the study demonstrate benefit.

### Design

A total of 12 patients and 12 carers will be recruited for the qualitative component from those allocated to treatment across all sites. Although eligibility of participants for the main study will have been determined through screening for the trial those selected for interview will reflect the diversity of the MND population. This will involve purposively selecting patients to reflect the variation within the predefined patient prognostic factors. It is anticipated that MND patients will not be able to tolerate an interview in addition to follow up assessments at local sites therefore an experienced research fellow will conduct the interviews at a time and location that is convenient for participants.

Ideally the patients and carers will be interviewed separately because they may have different views but joint interviews will be undertaken if requested by the participants. The research team are experienced in working with MND patients and appreciate the need for sensitivity whilst conducting the qualitative interviews with these vulnerable participants.

### Conduct

The in-depth interviews will be undertaken with DP patients 1 and 6 months post implantation. The first interview at 1 month will focus on the intervention and the practicalities of having the implant fitted and adjusting to life using the device. This information will be essential to inform the clinical team of issues related to both understanding the procedure including use of the equipment and any beneficial or adverse impact it may have.

A second interview will be undertaken at approximately 6 months post implantation. This will focus on the impact the intervention has had on QoL. Changes to QoL are reported to occur within this timeframe for all MND patients. Six months is also considered an appropriate time to allow patients receiving DP (and caregivers) to become familiar with the intervention and its impact on QoL. Interviews will provide an opportunity to take account of their views and experience of the intervention.

### Analysis

Data from the 48 qualitative interviews will be recorded, transcribed and undergo Framework analysis [22]. Although Framework analysis was developed for applied policy it has proved useful in applied health research. Analysis will be ongoing and iterative involving concurrent data collection and analysis, with systematic efforts to check and refine developing categories of data. Themes identified in the early phases of data collection will inform the areas of investigation in later interviews. The emerging analysis will be discussed at regular team meetings to identify recurring themes within the data which will explore respondents’ underlying reasoning, discuss deviant cases and reach agreement on recurrent themes and findings.

### Data collection

Once participants have been enrolled and allocated their treatment the data collection process starts (Table 1). Data collection occurs at baseline, 2, 3, 6, 9 and 12 months for both groups. Additionally the DP group will undergo data collection at the time of surgery and 1 week following surgery. The subgroup of 12 participants and carers who will be undertaking the qualitative sub study will also undergo interviews at 1 and 6 months post implantation.

**Table 1:** Data collection

**Data collection tool**	**Time point of study**	**When collected/given to patient**	**By who**	**Purpose**
Informed consent form	Recruitment	In clinic, face to face	Neurology or respiratory consultant	Ensure participants have been consented appropriately
Screening and eligibility assessment form	Recruitment/Screening	In clinic	Neurology or respiratory consultant or research nurse	Ensure protocol violations or deviations are avoided. Include ECG, blood gases, blood test, FVC and phrenic nerve evaluation tests
ALSFRSr	Screening	As above	As above	Allows minimisation on bulbar function
Survival	1 week, 2, 3, 6, 9, 12 months, then finally at last follow up for last patient	In clinic, telephone	Research nurse	Primary outcome measure
*EQ5D questionnaire (patient and carer)	Screening, 2, 3, 6, 9 and 12 months	In clinic or over the phone	Neurology or respiratory consultant or research nurse	QALYs, secondary outcome measure
SF36	Screening, 2, 3, 6 and 12 months	As above	As above	Generic quality of life, secondary outcome measure
Sleep Apnoea Quality of Life (SAQLI)	Screening, 2, 3, 6 and 12 months	As above	As above	Respiratory specific quality of life, secondary outcome measure
*Caregiver Burden Inventory questionnaire	Screening, 2, 3, 6 and 12 months	As above	As above	Secondary outcome measure
Side effects/ adverse event/concomitant medications and devices forms	All time points as required	As above	As above	AE/SAEs
Healthcare resource use	2, 3, 6, 9 and 12 months	As above	As above	Economic, secondary outcome measure
Patient Diary incorporating:	1 week, 2, 3, 6, 9 and 12 months	In clinic, at hospital or at home	Neurology or respiratory consultant and Patient and Carer	Main outcome Record DP and NIV use
* NIV use				
* DP use and				
* DP Parameters setting				
NIV use	Screening	As above	As above	Main outcome
Medical history and examination on CRF	Screening and 12 months	In clinic	Neurology or respiratory consultant	Eligibility for trial, safety
Surgery evaluation form/ pre op safety check	Screening, Surgery and 1 week	In clinic or hospital	Neurology or respiratory consultant or Surgeon	Safety and eligibility for surgery
Surgical implantation/ intra operative form	Surgery	In hospital	Neurology consultant or Surgeon	Testing DP device in situ
Discharge evaluation form	Surgery	In hospital	Neurology or respiratory consultant or surgeon	Demonstrate patient and carer competent to use and care for DP device
DP parameters setting	Surgery	Clinic	Neurology or respiratory consultant	Evaluate the DP device, allow optimal use of device
*Qualitative interview (n = 12, Patient and carer)	1 and 6 months post implantation	Participants place of choice	Qualitative fellow	Sub study outcome

### Trial database - prospect

Prospect is a CTRU online Database system. Users are able to perform data entry remotely via their secure account, access to which is restricted by use of an individual username and password. The study manager, data managers, PI’s, co-investigators, research nurses and administrators will have access to relevant anonymised data. Data input will be the responsibility of the research nurses. The system has a full electronic audit trail and will be regularly backed up. The secure data management system will incorporate quality control procedures to validate the study data. Error reports will be generated where data clarification is needed. Data quality will be the responsibility of the Sheffield CTRU Trial Manager and the Data Management Team.

### Data handling and confidentiality

Detailed data management and data quality issues will be set out in a data management and monitoring plan. Data will be collected and retained in accordance with the Data Protection Act 1998. Anonymised trial data will be entered onto a validated database system designed to an agreed specification between the Chief Investigator and Sheffield CTRU. Output for analysis will be generated in a format and at intervals to be agreed between Sheffield CTRU and the Chief Investigator. Trial documents will be retained in a secure location during and after the trial has finished.

All source documents will be retained for a period of at least 5 years following the end of the trial. Where trial related information is documented in medical records those records will be retained for at least 5 years after the last patient last visit.

Monitoring and audit by the relevant health authorities will be permitted by the sponsor. These include the Research Ethics Committee and local R&D departments. The sponsor will be allowed to monitor and audit the trial at each site and be allowed access to source data and documents for these purposes.

### Adverse events

All adverse events will be reported in accordance with the CTRU Adverse Event and Serious Adverse Events (SAE) SOP (PM004) which incorporate the Medicines for Human Use (Clinical Trials) Regulations 2004 (SI 2004/1031) definitions. All trial participants will be encouraged to contact and inform their site research team if they experience any of the medical problems outlined under SAE’s or relevant AE’s included (above). Those that are not picked up through general contact will be identified at follow up visits. A member of the site study team will enquire about any adverse events since the previous visit and record these on the adverse event paper CRF and database. For any SAEs a paper and database entry will be completed. The event will be assessed by the local Principal Investigator and the form will be kept in the site file. SAEs will be reported in the periodic safety reports to the research ethics committee. Standard or expected disease progression is an SAE exclusion. Adverse events which must be reported include chest infection requiring the use of antibiotics, infection at the DP site or a revision of the DP device.

### Statistics

#### Sample size

The sample size calculation is based on log-rank test, using Simpson’s rule as implemented in Stata version 11.1 [23] to allow for the unequal length of follow-up [24]. The study duration comprises an 18-month recruitment period and a 12-month follow-up period, giving a maximum follow-up of 30 months and a minimum of 12 months. Assuming control group survival proportions of 45%, 20% and 10% at the minimum, average and maximum follow-up times respectively, a hazard ratio of 0.45 and an additional 10% loss-to-follow-up, a total of 108 patients (54 per group) are needed to ensure a power of 85% using a two-sided type I error of 5%. The control group figures are conservative estimates based on the sole randomised controlled trial of NIV, which is now considered standard care in the UK. The FDA study of DP in ALS/MND has estimated a one year survival of 86% after study entry for patients using DP and NIV (personal communication). We have estimated the sample size on a conservative (but clinically important) 1-year difference in survival of 45% versus 70%, which produces the estimated hazard ratio of 0.45. It is anticipated that we will have complete survival data on all subjects recruited, based on previous experience in MND trials. With regard to quality of life data we anticipate a low level of missing data due to loss to follow up. We have reviewed the patients who were initiated on NIV in the year up to Jun 2009 at the study sites and we have maintained contact with 100% of those patients surviving at 12 months. The appointment of a research nurse at each study site will enable home visits if necessary to collect the quality of life data. We have however allowed for a 10% loss to follow up in the sample size/power calculation.

### Data analysis

The primary outcome is overall survival, defined as the duration from randomisation to death. This will be analysed by Cox regression, with covariates including treatment group and the minimisation factors. As a secondary analysis we will also report survival separately for patients who are NIV tolerant and those who are NIV intolerant. The proportionality assumption will be assessed using time-dependent covariates and scaled Schoenfeld residuals [25]. The change from baseline for QoL outcomes will be analysed by two methods. The first analysis will compare the change from baseline at 12-months using analysis of covariance (ANCOVA) in which the treatment group and the baseline score are included as covariates along with minimisation factors. The second analysis will assess the QoL over the entire 12-month period by modelling the change from baseline by repeated measures ANCOVA with the same covariates. QoL will be summarised both with imputation for missing data (in particular, assigning a score of zero following the date of death) and without. The safety and tolerability profiles will be reported by analysing the proportion of patients experiencing adverse outcomes. A description of the statistical analysis of efficacy and safety outcomes will be written in the trial Statistical Analysis Plan by the trial statistician.

### Economic evaluation

A cost-utility analysis will be undertaken using the costs, EQ-5D and mortality data from trial. The analysis will take a NHS and Personal Social Services (PSS) perspective, with an additional analysis that incorporates carer QALYs within the incremental cost-effectiveness ratio (ICER). This will be supplemented with decision analytic modelling to estimate lifetime cost-effectiveness for the patient cohort recruited to the trial. Resource use for insertion of the pacing system – theatre time, ward stay and any critical care - will be gathered from theatre and patient administration system (PAS) records. Resource use relating to NIV and other NHS and PSS services will be collected at all follow-up visits. Unit costs for insertion will be based on hospital unit costs. Market prices will be used for the pacing system and its associated costs, with an equivalent annual cost being calculated based on the lifespan of the system based on past experience. NIV costs will be based on business case and contracting information relating to existing NIV services within the participating centres. Other unit costs will be taken from the most recent National Reference Costs, British National Formulary and PSSRU publication ‘Unit costs of health and social care’. The EQ-5D will be completed at baseline and all follow-up visits by patients and the main carer of the patient. QALYs will be estimated using straight line interpolation between data points. Both costs and QALYs will be discounted at 3.5% per annum. Mean incremental costs and QALYs will be combined into an ICER, and sampling uncertainty represented by plots on the cost-effectiveness plane and associated cost-effectiveness acceptability curves (CEACs).

Missing data will be imputed using multiple imputation. An additional analysis will incorporate carer QALYs within the ICER. A decision analytic model will be constructed that will be validated by replicating the results of the trial, and then results extrapolated to conduct a lifetime analysis. Extrapolation will use transition probabilities estimated from a survival analysis based on the 12 month follow-up data from the trial. Probabilistic sensitivity analysis will be undertaken, with further deterministic analyses using SF-6D utilities and including carer utilities. The feasibility of a mixed treatment comparison that includes DiPALS, the study by Bourke et al (2006) and the ongoing Synapse study will be assessed. This will form the basis of an additional cost-effectiveness analysis if a valid comparison can be undertaken. As with the trial based analysis, results will be presented in terms of an ICER and CEACs.

### Trial oversight

Three committees have been established to oversee the conduct of the study: Trial Management Group (TMG), Trial Steering Committee (TSC) and Data Monitoring and Ethics Committee (DMEC). All committees are governed by Sheffield CTRU standard operating procedures. The TMG consists of the Chief and Principal Investigators and key staff within the CTRU. The role of the TMG is to implement all parts of the trial and to act on the recommendations from the TSC and DMEC.

The TSC consists of the Chief Investigator, key staff within the CTRU (as non voting members), an independent chair and three independent members. The roles of the TSC are to provide supervision of the protocol and statistical analysis plan, provide advice on and monitor progress of the trial, to review information from other sources and to consider recommendations from the DMEC. The DMEC consists of an independent chair and 2 independent members including a statistician. The DMEC has responsibility for monitoring the results provided by the trial statistician to the plan described in the trial protocol with reference to efficacy and safety, reviewing information from other sources, providing recommendations to the TSC on why the trial might be modified or discontinued in terms of ethics and safety and considering adverse events. There are no planned interim analyses for the trial.

### Monitoring arrangements

Trial set up and monitoring arrangements have been agreed with the trial Sponsor (Sheffield Teaching Hospitals). Monitoring assessments are planned at site initiation and following the recruitment of a 3^rd^ patient at each site. Thereafter either an annual or a triggered visit will be performed at sites to monitor their performance and perform source data verification and data completeness checks. All research governance approvals and contracts were checked at site initiation prior to the initiation of recruitment.

### Ethical considerations

The trial received Research Ethics Committee favourable opinion in July 2011. Trial sites (Sheffield Teaching Hospitals, University Hospital South Manchester, Oxford Radcliffe Hospitals, Newcastle Upon Tyne Hospitals and Plymouth Hospitals NHS Trust) have locally approved the study via local (Trust specific) site assessment. All study documentation including approved patient information sheets, consent forms, CRF’s and questionnaires can be found in the local site file.

### Indemnity and insurance

This trial will be conducted in accordance with the Medicines for Human Use (Clinical Trials) Regulations (SI 2004/1031). The trial has been financed by the HTA and details have been drawn up in a separate agreement. This is an NHS sponsored study. If there is negligent harm during the clinical trial when the NHS body owes a duty of care to the person harmed, NHS indemnity will cover NHS staff, medical academic staff with honorary contracts and those conducting the trial. The University of Sheffield has in place insurance against liabilities for which it may be legally liable and this cover includes any such liabilities arising out of this clinical trial.

### Reporting and dissemination

Results of the trial will be disseminated in peer reviewed scientific journals and clinical and academic conferences. Details of the trial will also be made available on the study website. Summaries of the research will be updated periodically to inform readers of the ongoing progress.

## Discussion and conclusion

This DiPALS study protocol will allow us to determine if Diaphragm Pacing improves participant survival and results in improved quality of life outcomes. Patient recruitment began in December 2011 and the last patient is expected to complete the trial in August 2014. The study report will be written up by March 2015.

## Competing interests

The authors declare that they have no competing interests.

## Synapse biomedical

Synapse Biomedical have provided support by providing devices and technical support for an initial case series and the ongoing RCT of Diaphragm Pacing. For the case series Synapse provided 2 of the 4 devices and for the RCT Synapse are providing 32 of the 54 devices at no cost. Synapse Biomedical have not been part of the design of the study and have no role in the study other than their standard technical support at implantation and for device/electrode malfunction.

## NIHR HTA

Grant awarded 2011 for 4 years (£1,010,000) to run the RCT of diaphragm pacing in ALS (DiPALS).

## *The Motor Neurone Disease Association and Henry Smith Charity*

Grant awarded in 2011 for 2 years (The Motor Neurone Disease Association and Henry Smith Charity 300,000). To support DiPALS RCT with purchase of 22 devices.

## Authors’ contributions

CJM and CM are the current Chief Investigator and Trial Manager respectively for the DiPALS trial and have contributed to the protocol. CLC is the Director of the CTRU supporting the trial and has contributed to the protocol. WOB, MJB and SD have contributed to the protocol particularly with respect to expertise in qualitative, statistical and health economic elements of the trial design respectively. RA, AB, SBa, SBi, SB, JE, SG, DK, NM, KM, NMu, JS, KT, TW and PJS are all PIs, Surgeons and co investigators on the trial and have contributed to the protocol. All authors contributed, read and approved the final manuscript.

## Pre-publication history

The pre-publication history for this paper can be accessed here:

http://www.biomedcentral.com/1471-2377/12/74/prepub

## References

[B1] The Scottish Motor Neuron Disease Register A prospective study of adult onset motor neuron disease in Scotland. Methodology, demography and clinical features of incident cases in 1989 J Neurol Neurosurg Psychiatry 1992 55 536 541 1640227 10.1136/jnnp.55.7.536 PMC489161

[B2] O’Toole O Traynor BJ Brennan P Sheehan C Frost E Coor B Epidemiology and clinical features of amyotrophic lateral sclerosis in Ireland between 1995 and 2004 J Neurol Neurosurg Psychiatry 2008 79 30 32 10.1136/jnnp.2007.117788 17634215

[B3] Worms PM The epidemiology of motor neuron diseases: a review of recent studies J Neurol Sci 2001 191 3 9 10.1016/S0022-510X(01)00630-X 11676986

[B4] McDermott CJ Shaw PJ Diagnosis and management of motor neurone disease BMJ 2008 336 658 662 10.1136/bmj.39493.511759.BE 18356234 PMC2270983

[B5] Turkington PM Elliott MW Rationale for the use of non-invasive ventilation in chronic ventilatory failure Thorax 2000 55 417 423 10.1136/thorax.55.5.417 10770824 PMC1745740

[B6] Bourke SC Noninvasive ventilation in ALS: indications and effect on quality of life Neurology 2003 61 171 177 10.1212/01.WNL.0000076182.13137.38 12874394

[B7] Fallat RJ Spirometry in amyotrophic lateral sclerosis Arch Neurol 1979 36 74 80 10.1001/archneur.1979.00500380044004 420626

[B8] Stambler N ALS CNTF Treatment Study Group Prognostic indicators of survival in ALS Neurology 1998 50 66 72 9443459 10.1212/wnl.50.1.66

[B9] Bourke SC Effects of non-invasive ventilation on survival and quality of life in patients with amyotrophic lateral sclerosis: a randomised controlled trial Lancet Neurol 2006 5 140 147 10.1016/S1474-4422(05)70326-4 16426990

[B10] DiMarco AF Onders RP Ignagni A Kowalski KE Mortimer JT Phrenic nerve pacing via intramuscular diaphragm electrodes in tetraplegic subjects Chest 2005 127 671 678 10.1378/chest.127.2.671 15706014

[B11] Onders RP Dimarco AF Ignagni AR Mortimer JT The Learning curve for investigational surgery: lessons learned from laparoscopic diaphragm pacing for chronic ventilator dependence Surg Endosc 2005 19 5 633 637 10.1007/s00464-004-8934-6 15776209

[B12] Levine S Rapid disuse atrophy of diaphragm fibers in mechanically ventilated humans N Engl J Med 2008 358 1327 1335 10.1056/NEJMoa070447 18367735

[B13] Onders R Complete worldwide operative experience in laparoscopic diaphragm pacing: results and differences in spinal cord injured patients and amyotrophic lateral sclerosis patients Surg Endosc 2009 23 1433 1440 10.1007/s00464-008-0223-3 19067067

[B14] HDE Summary of Safety and Probable Benefit (SSPB) FDASeptember 28 2011 H100006b http://www.accessdata.fda.gov/cdrh_docs/pdf10/H100006b.pdf

[B15] FDA approval letter September 28 2011 http://www.fda.gov/MedicalDevices/ProductsandMedicalProcedures/DeviceApprovalsandClearances/Recently-ApprovedDevices/ucm278684.htm

[B16] Lechtzin N Early use of non-invasive ventilation prolongs survival in subjects with ALS Amyotroph Lateral Scler 2007 8 3 185 188 10.1080/17482960701262392 17538782

[B17] Onders RP Amyotrophic lateral sclerosis: the Midwestern surgical experience with the diaphragm pacing stimulation system shows that general anesthesia can be safely performed Am J Surg 2009 197 386 390 10.1016/j.amjsurg.2008.11.008 19245920

[B18] Brooks R EuroQol: the current state of play Health Policy 1996 37 53 72 10.1016/0168-8510(96)00822-6 10158943

[B19] Novak M Guest C Application of a multidimensional caregiver burden inventory Gerontologist 1989 29 798 803 10.1093/geront/29.6.798 2516000

[B20] Britten N Qualitative research methods in general practice and primary care Fam Pract 1995 12 104 114 10.1093/fampra/12.1.104 7665030

[B21] Pope C Mays N Reaching the parts other methods cannot reach: an introduction to qualitative methods in health and health services research BMJ 1995 311 42 45 10.1136/bmj.311.6996.42 7613329 PMC2550091

[B22] Ritchie J Spencer L Bryman A, Burgess R Qualitative data analysis for applied policy research Analysing qualitative data 1994 Routledge, London

[B23] StataCorp Stata Statistical Software: Release 11 2009 StataCorp LP, College Station, TX

[B24] Schoenfeld DA Sample-size formula for the proportional-hazards regression model Biometrics 1983 39 499 503 10.2307/2531021 6354290

[B25] Therneau TM Grambsch PM Modeling survival data: extending the Cox model 2010 Springer, New York

